# Pepper mild mottle virus: Agricultural menace turned effective tool for microbial water quality monitoring and assessing (waste)water treatment technologies

**DOI:** 10.1371/journal.ppat.1007639

**Published:** 2019-04-18

**Authors:** Erin M. Symonds, Karyna Rosario, Mya Breitbart

**Affiliations:** College of Marine Science, University of South Florida, Saint Petersburg, Florida, United States of America; University of Michigan Medical School, UNITED STATES

## Introduction

Domestic wastewater pollution in environmental waters or water reuse supplies represents a threat to public health because of high concentrations of diverse pathogens associated with human excreta [1]. Since it is difficult to directly measure waterborne pathogens of concern, microbial water quality monitoring efforts often use surrogates or indicator organisms that are easily detected and whose presence reflects pathogen persistence [2]. Here, we describe an unconventional viral indicator of wastewater pollution, pepper mild mottle virus (PMMoV), a plant pathogen that was first proposed as a water quality indicator in 2009 [3] and promises to improve microbial water quality management worldwide [4].

## Why is PMMoV found in human feces and wastewater?

**Fig 1:**
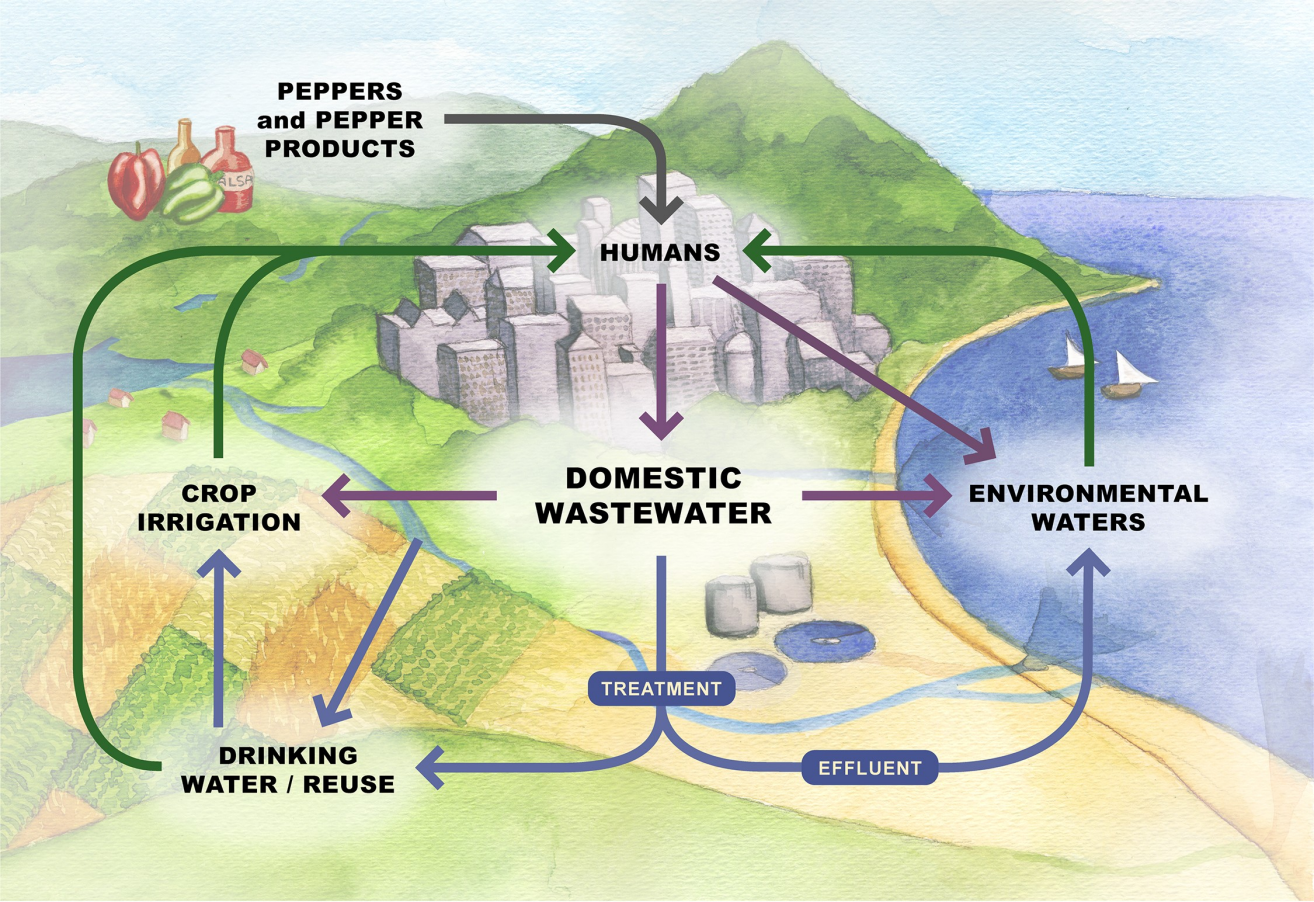
The plant pathogen PMMoV has emerged as a useful viral indicator of wastewater pollution because of its presence throughout the domestic wastewater cycle. PMMoV is excreted from humans in high concentrations after consumption of processed food products, such as hot sauces, that contain infected peppers (gray arrow). Because PMMoV is consistently found at high concentrations in domestic wastewater, it can be used as a marker to trace untreated (purple arrows) or treated (blue arrows) wastewater in the environment. Humans are commonly exposed (green arrows) to water and food resources affected directly or indirectly by wastewater discharges through the consumption of drinking water, fresh agricultural produce, or shellfish raised in polluted environmental waters, as well as the accidental ingestion of environmental water (e.g., lakes, reservoirs, rivers, coastal waters). The viral surrogate PMMoV reflects the presence and persistence of human enteric viruses in such resources, serving as a vital tool for monitoring microbial water quality, (waste)water treatment efficiency, and food safety. *Artwork by Anne Martin (hungrybraindesign*.*com)*. PMMoV, pepper mild mottle virus.

Considering that most recognized viral pathogens causing human gastrointestinal disease contain RNA genomes [5], early metagenomics studies sought to characterize the RNA viral communities (i.e., viromes) in the feces of healthy individuals [6]. Unexpectedly, these viromes were dominated by sequences similar to plant-infecting viruses that are acquired through dietary consumption of plant products [6]. By far, the most abundant RNA virus identified in the feces of healthy individuals was PMMoV [6], which has also been readily identified in untreated wastewater from numerous locations (summarized in [4, 7]; Fig 1). PMMoV infects various species of peppers (*Capsicum* spp.), and its presence in human feces originates from the consumption of infected peppers, which are frequently used in processed pepper products (e.g., hot sauces, curry sauces, dry spices; Fig 1) [6, 8, 9]. Up to 10^8^ PMMoV gene copies are found in a single milliliter of hot sauce [9], and healthy individuals can excrete up to 10^9^ gene copies in 1 gram of feces [6]. Although the presence of PMMoV in human feces presumably depends on dietary consumption of pepper products, which in turn may depend on age and food preferences of a given individual, PMMoV appears to be widespread and globally distributed, given that this virus has been consistently detected in untreated wastewater from Africa, the Americas, Asia, Australia, and Europe (summarized in [4, 7]). PMMoV can also be detected in treated wastewater effluent and in environments impacted by wastewater discharges (treated or untreated; Fig 1).

## How can we take advantage of PMMoV as an indicator of wastewater pollution?

Microbial surrogates used to characterize fecal pollution in environmental monitoring programs traditionally use fecal indicator bacteria (FIBs; e.g., *Escherichia coli* and enterococci), a group of bacteria that are native to the guts of animals. FIBs are the most affordable and commonly used fecal pollution indicators in the world. Unfortunately, FIB presence does not always correlate with human pathogens and/or human health risks, especially in the case of human enteric viruses [10–12]. Moreover, the detection of FIBs may not accurately reflect fecal contamination because of their ecology and extraintestinal reservoirs in soil and sediment [10, 13]. Another strategy for tracking fecal pollution is to monitor for specific reference viral pathogens of interest, such as norovirus and adenovirus. However, analytical tools for detecting reference enteric viruses are expensive, laboratory intensive, and often have efficiencies less than 10% [14, 15]. In addition, enteric viruses are typically found in low concentrations (<10^5^ copies per liter) in domestic wastewater in the absence of an outbreak [16] and further diluted upon discharge to environmental waters, which hinders their detection [14, 15].

Because viral enteric pathogens are not practical indicators of fecal pollution, viruses infecting gut bacteria have also been investigated. Viruses that infect FIBs, such as male-specific and somatic coliphages, are the most often used viral surrogates of fecal pollution; however, they do not always correlate with the presence of enteric viruses in the environment or during treatment processes (summarized in [14]). CrAssphage, a virus that infects human gut bacteria (*Bacteroides intestinalis*), was recently highlighted as a promising viral surrogate of human fecal pollution [17, 18]. Studies have found that it is highly human specific and is found in similar concentrations as the microbial source-tracking marker *Bacteroides* HF183 in wastewater and in polluted environmental waters. Future research is needed to understand the potential applications of this novel indicator, how it correlates with human pathogens, and its advantages over existing microbial surrogates of fecal pollution, including PMMoV.

PMMoV is consistently detected in domestic wastewater throughout the world, often at higher concentrations than human enteric pathogens; thus, it has been used as a viral surrogate to identify fecal pollution in the environment (summarized in [4, 7]; Fig 1). PMMoV is a unique viral indicator that offers several advantages over traditional indicators. First, concentrations of up to 10^10^ PMMoV gene copies per liter of domestic wastewater circumvent traditional methodological limitations associated with molecular detection of viruses in low concentrations, thus facilitating monitoring efforts. Although PMMoV concentrations vary over space and time, 10^6^ to 10^10^ gene copies per liter of domestic wastewater are consistently detected. Second, PMMoV’s dietary origin means that its concentration in domestic wastewater does not depend on active viral infection in the human population. This is an important consideration because it is not practical to regularly test for all known enteric pathogens, which exhibit different infection dynamics and seasonality. Third, unlike FIBs, PMMoV has not been detected in environmental waters free of fecal pollution and is typically not found in animals other than humans. However, PMMoV concentrations much lower than those found in domestic wastewater have been reported from birds (chickens, geese, swans, and seagulls), dogs, and cows (summarized in [4, 7]; [19]). Consequently, the use of PMMoV as a human-specific indicator for source-tracking purposes needs further evaluation. Nevertheless, PMMoV has been recognized as a promising viral indicator of domestic wastewater, with detection in environmental waters that is even comparable to nonbiological surrogates of wastewater contamination (e.g., caffeine, pharmaceuticals; summarized in [4, 7]).

## Is PMMoV detection indicative of health risks associated with exposure to wastewater pollution?

Ideally, the detection of a surrogate organism should correlate with health risks associated with using water resources impacted by wastewater. The high concentration of PMMoV in treated domestic wastewater effluent and high detection rates in environmental waters originally led to PMMoV being proposed as an ultraconservative wastewater indicator, whose detection may not correlate with enteric pathogens or health risks [3, 20, 21]. However, PMMoV detection and quantification has now been associated with human enteric pathogens and health risks exceeding benchmark limits [22–25]. Furthermore, PMMoV persistence in seawater and river water is comparable to that of human enteric viruses, ranging from 7 to 21 days, depending upon water temperatures [3, 24]. Data combined from multiple studies investigating PMMoV detection in parallel with human pathogens in environmental waters (groundwater, seawater, and surface freshwater) revealed that PMMoV was detected consistently (94.2% of samples) and co-occurred with enteric pathogens in the majority (72.3%) of tested samples [4]. Notably, PMMoV was most often detected along with human pathogens in waters exposed to untreated domestic wastewater and least often in the absence of known pollution sources (11.4% PMMoV-positive samples, with 0% pathogen co-occurrence). Therefore, PMMoV seems to be a conservative tracer, but its detection and concentration correlate with the presence of human enteric pathogens in environmental waters exposed to wastewater contamination.

PMMoV detection and quantification also correlated with health risks associated with recreation in contaminated environmental waters. In coastal waters contaminated with treated and untreated domestic wastewater, PMMoV detection corresponded to health risks greater than the United States Environmental Protection Agency (US EPA) health benchmark for safe recreation [22, 23]. For coastal waters with untreated domestic wastewater pollution, PMMoV concentrations greater than 5.4 × 10^3^ gene copies per liter corresponded to health benchmark exceedance [23]. In contrast, for coastal waters exposed to secondary treated domestic wastewater pollution, 1.8 × 10^3^ PMMoV gene copies per liter corresponded to the US EPA health benchmark for safe recreation. Future research is needed to better understand the correlations between PMMoV, enteric pathogens, and health risks in other environmental water matrices (e.g., freshwater), in different climates, and in the presence of tertiary treated wastewater.

## Can PMMoV be used to evaluate the efficiency of water treatment processes?

To protect public health as well as to ensure the microbial safety of drinking water, disinfection treatment processes need to reduce virus concentrations in wastewater prior to direct water reuse or discharge into environmental waters (Fig 1) [12, 26]. It is often difficult to determine the log reduction of viruses achieved by a given treatment technique because many viruses are found in concentrations too low to quantify and human enteric viruses of public health concern, such as norovirus, lack culture-based methods or are difficult to culture. Consequently, molecular methods such as quantitative reverse transcription PCR are commonly used to test virus reductions. In these contexts, PMMoV has proven to be an ideal virus process indicator to evaluate drinking water, wastewater, and water reclamation treatment technologies and facilities because, unlike other viruses, its high concentrations in wastewater allow for the quantification of virus gene copy removal at full-scale treatment plants [27–30]. Moreover, PMMoV reduction levels during treatment of wastewater [16, 30, 31] and drinking water are usually comparable to those of human enteric viruses [27–29]. PMMoV gene copy removal more often has a significant, positive correlation with the gene copy removal of human enteric viruses in comparison with the gene copy removal of other viral indicators (bacteriophages MS2 and φX174) during drinking water treatment (at plants with coagulation-sedimentation, rapid sand filtration, ozonation, and biological activated carbon treatments) [28]. In addition to having practical applications for measuring virus reduction at the full-scale treatment facilities, PMMoV is also useful for quantifying virus reductions by innovative water treatment technologies at smaller scales (summarized in [4]) as well as point-of-use household drinking water treatment [32] and on-farm riverbank filtration systems [33]. Because PMMoV is currently quantified using molecular methods that cannot determine virus infectivity, the incorporation of culture-based analyses (which would require plant growth chambers) [8] and/or selective pretreatment for infectious particles could improve future virus reduction analyses to assess treatment efficiency [34].

## How can we better exploit PMMoV to safeguard public health?

In addition to being a viral indicator for water quality monitoring purposes, PMMoV holds potential as a surrogate for enteric viruses in the assessment of food safety (Fig 1). Although more investigations are needed to confirm the application of PMMoV in agricultural contexts, PMMoV has been a useful surrogate for enteric viruses on crops irrigated directly and indirectly with domestic wastewater [33, 35]. Specifically, PMMoV concentrations determined in irrigation water enabled subsequent quantitative microbial risk assessments, which in turn facilitated the evaluation of best practices and practical recommendations regarding safe water reuse strategies for consumers. Furthermore, PMMoV frequently co-occurred with human norovirus in shellfish collected from coastal areas [4] and was consistently detected in shellfish exposed to point source fecal pollution [19], suggesting that PMMoV may be a useful enteric virus surrogate to monitor the microbial quality of shellfish harvested for raw consumption.

From the serendipitous discovery that plant viruses dominate human feces, PMMoV has emerged as a unique surrogate for enteric viral pathogens with proven applications for monitoring microbial water quality, (waste)water treatment efficiency, and food safety (summarized in [4]). Currently, PMMoV is detected and quantified using virus concentration and molecular methods, which are often too time consuming and expensive for use in widespread monitoring. Future research is warranted to develop rapid, lab-free, inexpensive methods for PMMoV detection, such as “dipstick”-type approaches based on immunological assays [14]. Development of more accessible detection methods will enable further exploitation of PMMoV, traditionally considered an agricultural threat, as a vital tool for protecting public health.
